# 2,6-Bis(prop-2-yn­yloxy)naphthalene

**DOI:** 10.1107/S1600536808031772

**Published:** 2008-10-09

**Authors:** Li Yao, Ruo-Jie Tao

**Affiliations:** a School of Computer and Information Engineering, Henan University, Kaifeng 475001, Henan, People’s Republic of China; b Institute of Molecular and Crystal Engineering, College of Chemistry and Chemical Engineering, Henan University, Kaifeng 475001, Henan, People’s Republic of China

## Abstract

The title compound, C_16_H_12_O_2_, crystallizes with one half-mol­ecule in the asymmetric unit. The mol­ecule lies on an inversion centre, located at the mid-point of the naphthyl group. All non-H atoms are almost coplanar, with a mean deviation from the least-squares plane of 0.0536 (11) Å. Mol­ecules are linked into a three-dimensional framework by a combination of C—H⋯O and C—H⋯π(arene) hydrogen bonds.

## Related literature

For compound preparation, see: Burchell *et al.* (2006 ▶). For related structures, see: Zhang *et al.* (2008 ▶); Ghosh *et al.* (2007 ▶).
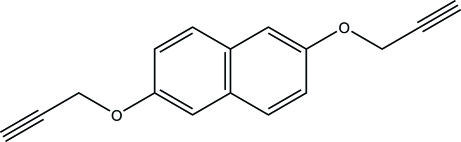


## Experimental

### 

#### Crystal data

C_16_H_12_O_2_
*M* *_r_* = 236.26
Orthorhombic, 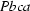

*a* = 7.5783 (11) Å
*b* = 8.0295 (12) Å
*c* = 20.972 (3) Å
*V* = 1276.1 (3) Å^3^
*Z* = 4
Mo *K*α radiation
μ = 0.08 mm^−1^
*T* = 293 (2) K
0.20 × 0.19 × 0.17 mm

#### Data collection

Bruker SMART APEXII CCD area-detector diffractometer
Absorption correction: multi-scan (*SADABS*; Bruker, 2005 ▶) *T*_min_ = 0.98, *T*_max_ = 0.99
6824 measured reflections
1250 independent reflections
952 reflections with *I* > 2σ(*I*)
*R*_int_ = 0.029

#### Refinement

*R*[*F* ^2^ > 2σ(*F* ^2^)] = 0.039
*wR*(*F* ^2^) = 0.099
*S* = 1.04
1250 reflections
82 parameters
H-atom parameters constrained
Δρ_max_ = 0.11 e Å^−3^
Δρ_min_ = −0.10 e Å^−3^

### 

Data collection: *APEX2* (Bruker, 2005 ▶); cell refinement: *APEX2*; data reduction: *SAINT* (Bruker, 2005 ▶); program(s) used to solve structure: *SHELXS97* (Sheldrick, 2008 ▶); program(s) used to refine structure: *SHELXL97* (Sheldrick, 2008 ▶); molecular graphics: *SHELXTL* (Sheldrick, 2008 ▶); software used to prepare material for publication: *SHELXTL*.

## Supplementary Material

Crystal structure: contains datablocks I, global. DOI: 10.1107/S1600536808031772/bg2213sup1.cif

Structure factors: contains datablocks I. DOI: 10.1107/S1600536808031772/bg2213Isup2.hkl

Additional supplementary materials:  crystallographic information; 3D view; checkCIF report

## Figures and Tables

**Table 1:** Hydrogen-bond geometry (Å, °) *Cg*1 and *Cg*2 are the centroids of the C4–C7/C7^i^/C8 and C4^i^–C7^i^/C7/C8^i^ rings, respectively.

*D*—H⋯*A*	*D*—H	H⋯*A*	*D*⋯*A*	*D*—H⋯*A*
C1—H1⋯O1^i^	0.93	2.56	3.385 (2)	148
C3—H3*A*⋯*Cg*1^ii^	0.97	2.76	3.579 (2)	143
C3—H3*A*⋯*Cg*2^iii^	0.97	2.76	3.579 (2)	143

Symmetry codes: (i) 
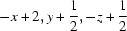
; (ii) 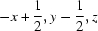; (iii) 
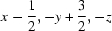
.

**Fig. 1.**
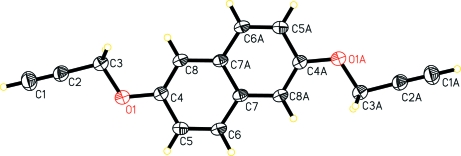
A view of the title compound, showing the atom-labeling scheme. Displacement ellipsoids are drawn at the 50% probability level and H atoms are shown as small spheres of arbritary radii. 'A' labeled atoms are generated by symmetry code -x + 2,-y + 1,-z.

**Fig. 2.**
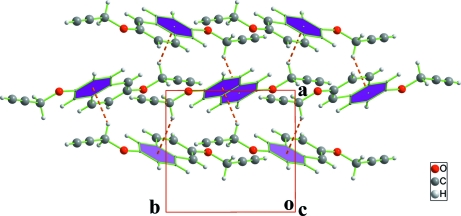
The three-dimensional supramolecular framework of the title complound formed by C—H···O and C—H···π(arene) hydrogen bonds.

## supplementary crystallographic information

### Comment

The molecule of the title compound (Fig. 1) lies on an inversion center, placed at the midpoint of the naphthyl group. Except for H atoms of the methylenes, all the remaining atoms are almost coplanar, with a mean deviation from the least-square plane to be 0.0675 (11) Å. The bond lengths and angles are normal.

No classical hydrogen bonds or π—π interactions are observed. The molecules of the title complex are linked into a three-dimensional framework by a combination of C—H···O and C—H···π(arene) hydrogen bonds (Fig. 2, Table 1). [*Cg*1 and *Cg*2 are the centroids of the C4—C7, C7^i^, C8 and C4^i^—C7^i^, C7, C8^i ^rings, respectively. Symmetry code: (i) -*x* + 2,-*y* + 1,-*z*.]

### Experimental

The title compound was obtaind unintentionally as the product of an attempted synthesis of a network complex (Burchell *et al.*,  2006) based on Co^II^ and 2,6-bis(prop-2-ynyloxy)naphthalene, by evaporation of a methyl alcohol and acetone solution of CoCl_2_, NaN_3_ and the title molecule, at 298 K. All chemical reagents were obtained commercially from Alfa Aesar Company and used without further purification.

### Refinement

All the H atoms could be detected in the difference electron density maps. Nevertheless, they were situated into the idealized position and refined using a riding model. C—H = 0.97 Å for the methylene groups and C—H = 0.93 Å for the remaining H atoms. *U*_iso_(H) = 1.2 *U*_eq_ (carrier C) for all the H atoms.

### Crystal data

**Table:**

C_16_H_12_O_2_	*F*(000) = 496
*M**_r_* = 236.26	*D*_x_ = 1.230 Mg m^−^^3^
Orthorhombic, *P**b**c**a*	Mo *K*α radiation, λ = 0.71073 Å
Hall symbol: -P 2ac 2ab	Cell parameters from 1948 reflections
*a* = 7.5783 (11) Å	θ = 2.7–26.2°
*b* = 8.0295 (12) Å	µ = 0.08 mm^−^^1^
*c* = 20.972 (3) Å	*T* = 293 K
*V* = 1276.1 (3) Å^3^	Block, colourless
*Z* = 4	0.20 × 0.19 × 0.17 mm

### Data collection

**Table:**

Bruker SMART APEXII CCD area-detector diffractometer	1250 independent reflections
Radiation source: fine-focus sealed tube	952 reflections with *I* > 2σ(*I*)
graphite	*R*_int_ = 0.029
φ and ω scans	θ_max_ = 26.0°, θ_min_ = 1.9°
Absorption correction: multi-scan (SADABS; Bruker, 2005)	*h* = −9→9
*T*_min_ = 0.98, *T*_max_ = 0.99	*k* = −9→9
6824 measured reflections	*l* = −12→25

### Refinement

**Table:**

Refinement on *F*^2^	Primary atom site location: structure-invariant direct methods
Least-squares matrix: full	Secondary atom site location: difference Fourier map
*R*[*F*^2^ > 2σ(*F*^2^)] = 0.039	Hydrogen site location: inferred from neighbouring sites
*wR*(*F*^2^) = 0.099	H-atom parameters constrained
*S* = 1.04	*w* = 1/[σ^2^(*F*_o_^2^) + (0.0447*P*)^2^ + 0.1717*P*] where *P* = (*F*_o_^2^ + 2*F*_c_^2^)/3
1250 reflections	(Δ/σ)_max_ < 0.001
82 parameters	Δρ_max_ = 0.11 e Å^−^^3^
0 restraints	Δρ_min_ = −0.10 e Å^−^^3^

### Special details

**Table:**

Geometry. All e.s.d.'s (except the e.s.d. in the dihedral angle between two l.s. planes) are estimated using the full covariance matrix. The cell e.s.d.'s are taken into account individually in the estimation of e.s.d.'s in distances, angles and torsion angles; correlations between e.s.d.'s in cell parameters are only used when they are defined by crystal symmetry. An approximate (isotropic) treatment of cell e.s.d.'s is used for estimating e.s.d.'s involving l.s. planes.
Refinement. Refinement of *F*^2^ against ALL reflections. The weighted *R*-factor *wR* and goodness of fit *S* are based on *F*^2^, conventional *R*-factors *R* are based on *F*, with *F* set to zero for negative *F*^2^. The threshold expression of *F*^2^ > σ(*F*^2^) is used only for calculating *R*-factors(gt) *etc*. and is not relevant to the choice of reflections for refinement. *R*-factors based on *F*^2^ are statistically about twice as large as those based on *F*, and *R*- factors based on ALL data will be even larger.

### Fractional atomic coordinates and isotropic or equivalent isotropic displacement parameters (Å^2^)

**Table:**

	*x*	*y*	*z*	*U*_iso_*/*U*_eq_	
O1	0.99206 (12)	0.82529 (11)	0.13702 (4)	0.0551 (3)	
C1	0.9300 (3)	1.1886 (2)	0.21293 (9)	0.0939 (7)	
H1	0.9416	1.2665	0.2455	0.113*	
C2	0.9155 (2)	1.09120 (19)	0.17226 (8)	0.0662 (5)	
C3	0.8995 (2)	0.97311 (17)	0.12002 (7)	0.0600 (4)	
H3A	0.7762	0.9479	0.1123	0.072*	
H3B	0.9492	1.0202	0.0814	0.072*	
C4	1.00020 (16)	0.70096 (16)	0.09247 (7)	0.0473 (3)	
C5	1.09064 (17)	0.55721 (17)	0.11300 (7)	0.0532 (4)	
H5	1.1365	0.5528	0.1541	0.064*	
C6	1.11120 (18)	0.42548 (16)	0.07337 (7)	0.0525 (4)	
H6	1.1699	0.3313	0.0881	0.063*	
C7	1.04565 (15)	0.42735 (15)	0.01009 (7)	0.0458 (3)	
C8	0.93214 (16)	0.70846 (16)	0.03204 (6)	0.0481 (4)	
H8	0.8713	0.8029	0.0187	0.058*	

### Atomic displacement parameters (Å^2^)

**Table:**

	*U*^11^	*U*^22^	*U*^33^	*U*^12^	*U*^13^	*U*^23^
O1	0.0576 (6)	0.0521 (6)	0.0555 (6)	0.0050 (5)	−0.0035 (5)	0.0030 (5)
C1	0.145 (2)	0.0720 (11)	0.0651 (12)	0.0012 (12)	0.0035 (12)	−0.0069 (10)
C2	0.0822 (11)	0.0563 (9)	0.0601 (10)	0.0052 (8)	0.0045 (8)	0.0045 (8)
C3	0.0666 (10)	0.0526 (8)	0.0609 (9)	0.0060 (7)	−0.0028 (7)	0.0032 (7)
C4	0.0400 (7)	0.0467 (7)	0.0553 (8)	−0.0026 (6)	0.0014 (6)	0.0045 (6)
C5	0.0497 (8)	0.0572 (8)	0.0525 (8)	0.0020 (7)	−0.0072 (6)	0.0088 (7)
C6	0.0476 (7)	0.0494 (7)	0.0603 (9)	0.0081 (6)	−0.0066 (6)	0.0109 (7)
C7	0.0364 (6)	0.0465 (7)	0.0546 (8)	−0.0008 (5)	−0.0014 (6)	0.0103 (6)
C8	0.0424 (7)	0.0446 (7)	0.0575 (9)	0.0042 (6)	−0.0021 (6)	0.0095 (6)

### Geometric parameters (Å, °)

**Table:**

O1—C4	1.3687 (16)	C5—C6	1.3541 (18)
O1—C3	1.4240 (16)	C5—H5	0.9300
C1—C2	1.162 (2)	C6—C7	1.417 (2)
C1—H1	0.9300	C6—H6	0.9300
C2—C3	1.454 (2)	C7—C8^i^	1.4136 (18)
C3—H3A	0.9700	C7—C7^i^	1.421 (2)
C3—H3B	0.9700	C8—C7^i^	1.4136 (18)
C4—C8	1.3695 (19)	C8—H8	0.9300
C4—C5	1.4098 (18)		
			
C4—O1—C3	117.34 (10)	C6—C5—C4	120.54 (13)
C2—C1—H1	180.0	C6—C5—H5	119.7
C1—C2—C3	178.25 (18)	C4—C5—H5	119.7
O1—C3—C2	108.28 (12)	C5—C6—C7	121.74 (12)
O1—C3—H3A	110.0	C5—C6—H6	119.1
C2—C3—H3A	110.0	C7—C6—H6	119.1
O1—C3—H3B	110.0	C8^i^—C7—C6	122.38 (12)
C2—C3—H3B	110.0	C8^i^—C7—C7^i^	120.34 (15)
H3A—C3—H3B	108.4	C6—C7—C7^i^	117.28 (16)
O1—C4—C8	125.63 (12)	C4—C8—C7^i^	119.98 (12)
O1—C4—C5	114.25 (12)	C4—C8—H8	120.0
C8—C4—C5	120.11 (13)	C7^i^—C8—H8	120.0
			
C4—O1—C3—C2	−176.80 (12)	C4—C5—C6—C7	0.9 (2)
C3—O1—C4—C8	2.36 (19)	C5—C6—C7—C8^i^	179.27 (12)
C3—O1—C4—C5	−178.77 (11)	C5—C6—C7—C7^i^	−1.0 (2)
O1—C4—C5—C6	−178.91 (12)	O1—C4—C8—C7^i^	178.05 (12)
C8—C4—C5—C6	0.02 (19)	C5—C4—C8—C7^i^	−0.75 (19)

Symmetry codes: (i) −*x*+2, −*y*+1, −*z*.

### Hydrogen-bond geometry (Å, °)

**Table:**

*D*—H···*A*	*D*—H	H···*A*	*D*···*A*	*D*—H···*A*
C1—H1···O1^ii^	0.93	2.56	3.385 (2)	148
C3—H3A···Cg1^iii^	0.97	2.76	3.579 (2)	143
C3—H3A···Cg2^iv^	0.97	2.76	3.579 (2)	143

Symmetry codes: (ii) −*x*+2, *y*+1/2, −*z*+1/2; (iii) −*x*+1/2, *y*−1/2, *z*; (iv) *x*−1/2, −*y*+3/2, −*z*.
